# Impact of inter-species hybridisation on antifungal drug response in the *Saccharomyces* genus

**DOI:** 10.1186/s12864-024-11009-3

**Published:** 2024-12-02

**Authors:** Federico Visinoni, William Royle, Rachel Scholey, Yue Hu, Soukaina Timouma, Leo Zeef, Edward J. Louis, Daniela Delneri

**Affiliations:** 1 Manchester Institute of Biotechnology, University of Manchester, Manchester, M1 7DN UK; 2 Division of Evolution and Genomic Sciences, School of Biological Sciences, Faculty of Biology Medicine and Health, University of Manchester, Manchester, M13 9PT UK; 3 Bioinformatics Core Facility, University of Manchester, Manchester, M13 9PT UK; 4 Phenotypeca Limited, BioCity Nottingham, Nottingham, NG1 1GF UK

**Keywords:** Yeast hybrids, Antifungal drug resistance, QTLmapping, Saccharomyces

## Abstract

**Background:**

Antifungal drug resistance presents one of the major concerns for global public health, and hybridization allows the development of high fitness organisms that can better survive in restrictive conditions or in presence of antifungal agents. Hence, understanding how allelic variation can influence antifungal susceptibility in hybrid organisms is important for the development of targeted treatments. Here, we exploited recent advances in multigenerational breeding of *hemiascomycete* hybrids to study the impact of hybridisation on antifungal resistance and identify quantitative trait loci responsible for the phenotype.

**Results:**

The offspring of *Saccharomyces cerevisiae* x *S. kudriavzevii* hybrids were screened in the presence of six antifungal drugs and revealed a broad phenotypic diversity across the progeny. QTL analysis was carried out comparing alleles between pools of high and low fitness offspring, identifying hybrid-specific genetic regions involved in resistance to fluconazole, micafungin and flucytosine. We found both drug specific and pleiotropic regions, including 41 blocks containing genes not previously associated with resistance phenotypes. We identified linked genes that influence the same trait, namely a hybrid specific ‘super’ QTL, and validated, via reciprocal hemizygosity analysis, two causal genes, *BCK2* and *DNF1*. The co-location of genes with similar phenotypic impact supports the notion of an adaption process that limits the segregation of advantageous alleles via recombination.

**Conclusions:**

This study demonstrates the value of QTL studies to elucidate the hybrid-specific mechanisms of antifungal susceptibility. We also show that an inter-species hybrid model system in the *Saccharomyces* background, can help to decipher the trajectory of antifungal drug resistance in pathogenic hybrid lineages.

**Supplementary Information:**

The online version contains supplementary material available at 10.1186/s12864-024-11009-3.

## Introduction

The tally of life-threatening infections associated with fungal pathogens is estimated to be on the same scale as tuberculosis and malaria with 13 million infections yearly, and a mortality of 1.5 million [1, 2]. The severity of fungal infection is exacerbated by the widespread occurrence of immunocompromising conditions such as cancer, human immunodeficiency virus (HIV) disease, tuberculosis and the coronavirus disease 2019 (COVID-19) [3]. Moreover, the rapid emergence of antifungal resistance in clinical strains, analogous to antibacterial resistance, has highlighted the need to expand the current toolset of treatment options targeting the main fungal pathogens, *Candida*, *Aspergillus* and *Cryptococcus* species. Specific strains of *Saccharomyces cerevisiae* are also opportunistic pathogens in immunocompromised patients [4].

The impact of the rise in antifungal resistance is now recognised as an emerging threat to public health and strategies are required to establish future mechanisms of combatting the risk caused by pathogenic fungi [5].

Currently, only four classes of antifungal drugs are in routine use: azoles, polyenes, pyrimidine analogues and echinocandins [6]. Azoles, such as fluconazole and miconazole, are the most commonly prescribed for the treatment of *Candida* and *Cryptococcus* infections [6, 7]. Azoles act as strong inhibitors of lanosterol demethylase (encoded by *ERG11*) disrupting ergosterol biosynthesis, an essential component of fungal membranes, leading to the accumulation of toxic sterol intermediates [8].

Amongst the pyrimidine analogues, flucytosine has been in use as an antifungal drug since 1968 [9] and is often used in combination with other antifungal agents to reduce occurrences of antifungal resistance [10]. Flucytosine is first converted in vivo in 5-fluorouridine by a cytosine deaminase and then phosphorylated to 5-fluoro-uridine-triophosphate (5-F-UTP) or reduced to 5-fluoro-deoxyuridine-monophosphate (5-F-dUMP). As 5-F-UTP the drug is incorporated into RNA, leading to the inhibition of protein synthesis. Instead, as 5-F-dUMP the antifungal act as an inhibitor of thymidylate synthase and, as a consequence, of purine and DNA synthesis [9, 11].

The most recent class of antifungal introduced for clinical use are echinocandins; lipopeptides which act through a non-competitive binding of the enzyme β-D-glucan synthase complex in both *Candida* an *Aspergillus spp.* Echinocandins, such as caspofungin and micafungin, inhibit the biosynthesis of β-D-glucan, a crucial component of the fungal cell wall, leading to osmotic stress and cell death [12].

The known molecular mechanisms of fungal resistance are often linked to mutations in the target gene or in the pathway affected by the drug. As a case in point, *Candida spp*. are known to develop resistance to fluconazole by the overexpression of the target gene, *ERG11*, or by the accumulation of point mutations which alter the structure of the molecular target. However, the high-level of resistance of clinical isolates can rarely be ascribed to the effect of single mutations, which, instead, is often attributable to a combination of traits and a gradual adaptation to the stressor [7, 13].

A population response to an environmental stressor is impacted by the genetic variation present within the gene pool, where there exists a spectrum of fitness based on allelic determination. This is true for all organisms including pathogenic fungi and can be associated to antifungal drug susceptibility. The strong selection pressures of exposure to such agents can result in the propagation of antifungal resistance [2], the emergence of fluconazole resistant *C. albicans* strains is well documented in long-term fluconazole treatment in HIV positive individuals [14]. Indeed, exposure to different concentrations of drugs can also influence the speed and nature of the adaptive response of pathogenic fungi and yeasts to antifungal drugs [15]. Therefore, to perform thorough investigations of the networks and pathways associated with antifungal resistance it is essential to carry out genome-wide investigations to sample the breadth of the cellular response to antifungal agents.

In recent years, a growing number of high-throughput and -omics studies have been successfully applied to the study of antifungal resistance and pathogenicity in both model systems, such as *Saccharomyces cerevisiae* and *Schizosaccharomyces pombe* [16–19], and pathogenic species such as *C. albicans* and *Cryptococcus neoformans* [20, 21]. However, such studies are often limited by the use of laboratory strains which precludes in-depth analysis into the mechanisms developing through adaptation and evolution in nature. To establish new routes of therapeutic treatment we require methods to identify genetic targets for antifungal drugs that consider the variation present across different species and natural populations of Ascomycota.

Moreover, numerous yeast pathogens are shown to have originated in a hybrid ancestor, undergoing divergence as a result of large-scale loss of heterozygosity (LOH) [22]. It has been reported that the clinically important opportunistic pathogen *C. albicans* derived from a single hybrid ancestor, and that *C. stellatoidea* and *C. africana* descended from the same ancestor and diverged as a consequence of large scale LOH [23]. The presence of *Candida* spp. hybrids with the potential of human virulence demonstrates the possibility that novel pathogenic lineages can arise as the result of hybridisation between species. Notably *C. orthopsilosis*, in which the hybrid demonstrates increased virulence over its parental lineages [24].

Inter-species hybrids within the *Cryptococcus* genus have been associated with pathogenesis and are estimated to contribute over 30% of cryptococcosis cases in Europe [25–27]. Hybrids between *C. neoformans* and *C. deneoformans* contribute the majority of hybridisation events in clinical environments [28] with a variable virulence compared to parent species [29]. Crucially *Cryptococcus neoformans* x *C. deneoformans* hybrids show evidence of transgressive segregation in traits related to antifungal drug resistance [21, 30].

As our understanding of the landscape of pathogenic yeast increases, it is clear hybridisation is a contributing factor in the adaption and evolution of the three main fungal pathogens, *Aspergillus*, *Candida* and *Cryptococcus* [22, 31, 32]. Thus, it is of importance to understand the role that hybridisation and hybrid genome plasticity have in driving antifungal resistance.

**Fig. 1:**
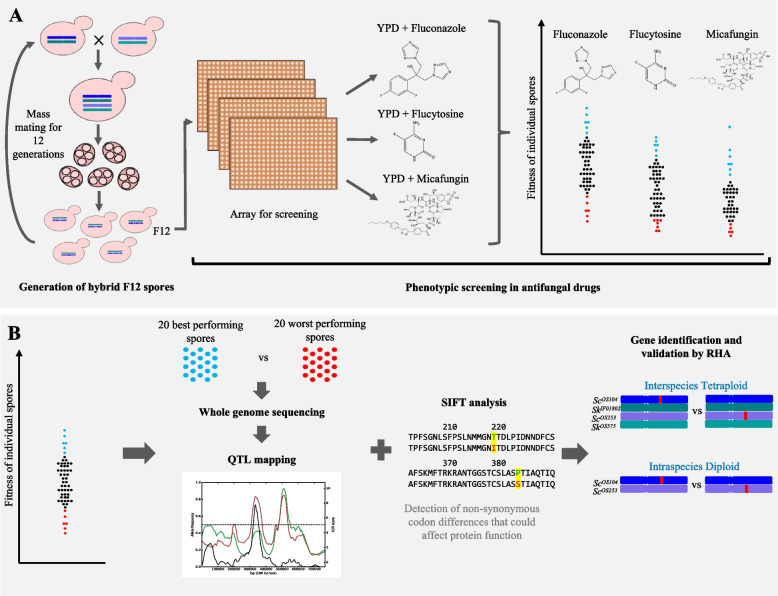
Experimental strategy for QTL studies in multigenerational inter-specific hybrids. **A** An F12 collection of interspecies hybrid progeny were generated, arrayed, and screened on YPD agar containing sub-lethal concentrations of antifungal drugs. Colony size was used as a proxy for fitness of the spores. **B** A pool of the top 20 performing and worst 20 performing offspring from each condition were sequenced and QTL regions, associated with antifungal susceptibility, identified. The QTL plot shows the frequency of the alternative allele (Sc^OS253^) in the high (red) and low (green) fitness pools, the black line represents the LOD score and the grey area the QTL region with the highest LOD score. Sorting Intolerant from Tolerant (SIFT) analysis allowed identification of candidate genes for validation via reciprocal hemizygosity analysis

In our previous work, we generated tetraploid hybrids between different strains of two species, namely *S. cerevisiae* and *S. kudriavzevii* (*Sc/Sk*). The tetraploids were able to undergo meiosis to produce diploid hybrid spores, which were randomly crossed and put through 12 meiotic cycles (F12) to allow ample recombination events [33]. Here, we have screened the F12 *Sc*/*Sk* diploid hybrid collection for growth on antifungal drugs. We applied an in-house pipeline for the identification of QTLs in complex hybrid background to unpick allelic variants associated with antifungal susceptibility (Fig. 1). *S. cerevisiae* and *S. kudriavzevii* are phylogenetically distinct species within the *Saccharomyces* sensu stricto complex, with a nuclear genetic divergence of ~ 20% [34], and their hybridisation has been observed in fermentation environments [35, 36]. The offspring revealed a broad phenotypic diversity in the majority of conditions tested, highlighting the large impact that gene interactions have on fitness. The QTL study identified hybrid-specific traits linked to resistant phenotypes in fluconazole, flucytosine and micafungin, highlighting how natural variations and hybridization could lead to resistant phenotypes. We also found genes that are drug specific and we identify 41 QTL regions that contain genes not currently associated with drug resistance in the published literature. The data generated represent a valuable resource for the identification of new markers and predictors of antifungal resistance and to inform drug development.

## Material and methods

### Strains used in the study

The parental tetraploid *S. cerevisiae* x *S. kudriavzevii* hybrid (Sc^OS253^/Sk^OS575^/Sc^OS104^/Sk^IF01802^) harbouring *S. kudriavzevii* mitochondria and 228 F12 diploid hybrid progeny generated in [33] were used in this study. Yeast strains were maintained in YPD medium (1% yeast extract, 2% peptone, 2% glucose, Formedium, UK) in 96 well-plates, and in PlusPlates (Singer Instruments, UK) with YPD + 2% agar incubated at 30 °C.

### Phenotypic tests

Diploid spores of *S. cerevisiae* x *S. kudriavzevii* hybrids were grown in YPDA at 30 °C and then inoculated into 100 µL YPD in a 96 well microtiter plate alongside the parental tetraploid (Sc^OS253^/Sk^OS575^/Sc^OS104^/Sk^IF01802^). The strains were incubated for 96 h at 30 °C and then sub-cultured in 384 well microtiter plate containing 70 µL YPD using the Singer Rotor HDA (Signer Instruments, Somerset, UK), prepared with four technical replicates of each strain.

For the phenotypic analysis of *S. cerevisiae* x *S. kudriavzevii* spores, the liquid cultures were grown to saturation at 30 °C and stamped in solid media plates at a density of 384 strains per plate. The spores were grown at 30 °C in YPDA and in YPDA in the presence of sublethal concentrations of antifungal drugs [37], specifically: YPDA + 5 μg/ml and 10 μg/ml of fluconazole, 10 μM and 20 μM of miconazole, 1 mg/ml and 2.5 mg/ml of caffeine, 25 ng/ml and 50 ng/ml of micafungin, 10 μg/ml and 20 μg/ml of flucytosine, 0.5 μg/ml and 1 μg/ml of bleomycin.

The plates were imaged with the PhenoBooth Colony Counter (Singer Instruments, UK) after 72 h of incubation and the size of the individual colonies was used as a proxy for fitness.

The median size from the four replicates of each spore was used in downstream analysis. To exclude outliers, the colony size of each spore was compared to the first and the third interquartile range of the four replicates. Spores with replicates of value higher than the third quartile or lower than the first quartile, by 125% of the interquartile range, were excluded.

### DNA Extraction and sequencing

Diploid spores of *S. cerevisiae* x *S. kudriavzevii* hybrids were inoculated in 1.5 mL of YPD and incubated overnight at 30 °C, shaking. Total DNA was purified using Epicentre Masterpure™ Yeast DNA Purification Kit (Lucigen, USA) and resuspended in 50 µL of RNase-free water. To remove any RNA contamination present in the sample, the purified DNA was incubated for 30 min at 37 °C with 1 µL of 5 µg/µL RNase A.

The quality of the purified DNA was assessed through gel electrophoresis on a 0.8% agarose gel and a Thermo Scientific™ NanoDrop Lite spectrophotometer (Thermo Scientific, UK). The DNA in each sample was quantified with a Qubit 4 Fluorometer (Thermo Scientific, UK).

### QTL mapping

The unmapped paired-end sequences from an Illumina HiSeq 4000 sequencer were quality assessed by FastQC [38]. Sequence adapters were removed, and reads were quality trimmed (to quality score q20) using Trimmomatic_0.36 [39]. The mapping, variant calling and Multipool analysis was performed as previously described [33]. Briefly, the reads were mapped against a reference hybrid genome containing the reference sequence for each founder species *S. cerevisiae* OS104 [40] and *S. kudriavzevii* IF0 1802 T Ultra-Scaffolds assembly [41] using BWA-MEM [42] (bwa_ 0.7.15). While working on this study, a newer genome assembly for *S. kudriavzevii* IFO1802T also became available [34]. Local realignment was performed with GATK_3.8.0 [43] and duplicates were marked with Picard Toolkit_2.1.0 (http://broadinstitute.github.io/picard/). The alignment quality was assessed with Qualimap_2.2.1 [44]. For each sample, variant calling was performed individually using Freebayes_1.1.0 [45] with ploidy setting at 1 and including the following parameters –minmapping-quality 30 –min-base-quality 20 –no-mnps. The resultant VCF files were filtered for 'type = SNP' variants and processed using R. Unique bi-allele markers for each founder species were identified. Reads depths below 10 were excluded. The parental allele counts in each F12 pool were then calculated by matching reference (RO) and alternate (AO) alleles to the bi-allele marker sets among the founders. The allele counts were provided to Multipool [46] and log_10_ likelihood ratios (LOD scores) were calculated across each chromosome. QTLs were identified and reported with an LOD support interval of 1, in regions where a minimum LOD score of 5 extended across at least 20 kb. The identified QTLs were annotated using the available annotation from *S. cerevisiae* OS104. Annotation for the *S. kudriavzevii* IF0 1810 T ultra-scaffolds assembly was performed using HybridMine [47] and can be found at https://github.com/Sookie-S/QTL_analysis_pipeline_hybrid_species/blob/main/Files/Genomes/GFF_files/.

The upstream pipeline including the multipool preparation and wrapper script and the pipeline for the QTL analysis can be found at https://github.com/Sookie-S/QTL_multi-locus_genetic_mapping_with_pooled_sequencing and https://github.com/Sookie-S/QTL_analysis_pipeline_hybrid_species/tree/main.

### Data analysis

Potential causal genes were analysed with the Sorting Intolerant from Tolerant (SIFT) algorithm to assess if amino-acids variants were predicted to influence the protein function. SIFT analysis were conducted using data from Bergstrom et al. 2014 [48] on the *S. cerevisiae* strains OS104 and OS253.

### Validation through RHA

Reciprocal hemizygosity analysis (RHA) provides a method of validation for identified, advantageous, alleles [49] and was broadly performed as in Naseeb et al. 2021 [33]. RHA was performed on candidate genes *BCK2* and *DNF1*. PCR-mediated deletion of the *S. cerevisiae* allele was performed in the F12 *Saccharomyces cerevisiae*/*Saccharomyces kudriavzevii* diploid hybrids (*Sc*^*OS104*^/*Sk*^*IF01802*^ and *Sc*^*OS253*^/*Sk*^*OS575*^) and the *S. cerevisiae* strains (*Sc*^*OS104*^ and *Sc*^*OS253*^). All deletion strains were confirmed using confirmation colony PCR (all primers used are included in Table S6). Mass mating was applied to generate reciprocal hemizygotes for both inter-species tetraploids and intraspecies diploids, hybrids were selected for on triple drug plates (300 μg/mL geneticin, 200 μg/mL nourseothricin and 15 μg/mL phleomycin).

The growth of *DNF1* and *BCK2* allele variants was tested in liquid YPD media with added flucytosine (50 μg/mL) and micafungin (50 ng/mL) on a FLUOstar Optima 466 plate reader (BMG) at 25 °C. The growth characteristics of the plate reader experiments were assessed with the R package Growthcurver using K as maximum biomass, r as maximum growth rate, auc_l as integral area and Tmid as the time at which the population density reaches 1/K. Statistics were performed using GraphPad Prism.

## Results and discussion

### Phenotypic screening of inter-specific hybrid offspring in the presence of antifungal drugs

Multigenerational diploid offspring of tetraploid hybrids [33] containing the genome of four strains belonging to two different species (Table 1), namely *S. cerevisiae* strain OS104 (*Sc*^*OS104*^) and OS253 (*Sc*^*OS253*^), and *S. kudriavzevii* strain IFO1802 (*Sk*^*IF01802*^) and OS575 (*Sk*^*OS575*^) were used to investigate growth changes in the presence of antifungals.

**Table 1:** Background and origins of all parental strains and hybrid tetraploids used for phenotypic screening

Species and hybrids	Strain	Origin
*Saccharomyces cerevisiae*	Sc^OS253^ (NRRL-Y12663)	Africa, Palm wine
	Sc^OS104^ (YPS128)	North American (woodland isolate)
*Saccharomyces kudriavzevii*	Sk^OS575^ (48BYC-4)	China, oak bark
	Sk^IFO1802^ (NCYC2889)	Japan, Decayed leaf matter
Sc/Sk Tetraploid hybrid	Sc^OS253^/Sk^OS575^/Sc^OS104^/Sk^IF01802^	DD's lab

High-throughput phenotyping on solid media [50] allowed us to assess how genotypic divergence of the hybrid spores impacts resistance or susceptibility to antifungal agents. The growth of the hybrid progenies along with their tetraploid parent was assessed in standard rich medium and in media containing inhibitors of ergosterol biosynthesis (fluconazole and miconazole), cell wall biogenesis (micafungin and caffeine) and nucleic acid biosynthesis (flucytosine and phleomycin). The antifungal drugs were added in the media at sub-lethal concentrations according to Gebre and co-workers [37] to allow for two-tailed selection to identify both high-performing and low-performing spores.

**Fig. 2:**
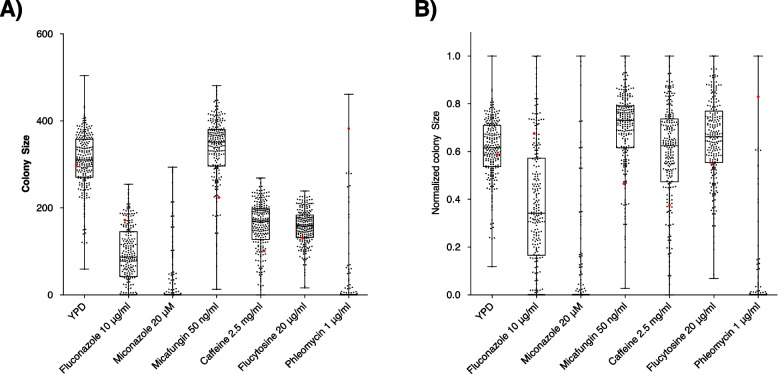
Box plot of the fitness of F12 diploid progeny for *S. cerevisiae/S. kudriavzevii* hybrids. Median colony size across four technical replicates is used as a proxy for fitness and calculated following incubation with different concentrations of antifungal drugs. Expressed as colony size (**A**) and as normalized colony size (**B**) parental tetraploid was used as a control and is highlighted in red. Each black dot represents a distinct F12 hybrid progeny. The upper and lower bars show the maximum and minimum values, the box represents the second and third quartile with the central line the median

The hybrid progeny exhibited a broad phenotypic space in all the six antifungal drugs screened (Fig. 2, Table S1, Figure S2). The higher dispersion was recorded in media with 10 μg/ml of fluconazole with a IQR (quartile coefficient of dispersion) of 0.55, compared to 0.14 in YPD (Table S1). The growth in miconazole and phleomycin, at 1 μg/mL, were characterised by low cell viability (< 99%), with only around 20% of the progeny able to sustain growth at the highest concentration tested (Table S1). The tetraploid parent control was able to grow in each of the antifungal drugs tested except for miconazole. Compared to its progeny, the parental tetraploid demonstrated a lower fitness than the median of the offspring in four out of six conditions (present in the upper quartile only in phleomycin and fluconazole; Fig. 2). This data highlights the transgressive traits of the hybrid offspring compared to the parent and supports the notion that the greater fitness of progeny may be the result of unlinking detrimental alleles present in the parental tetraploid.

The hybrid spores grown in media containing micafungin exhibited a higher median colony size compared to growth in YPD (Fig. 2A, Table S1, Figure S2). Previous studies observed an abnormal morphology in yeast cells treated with micafungin, reportedly similar to *HOC1* and *MNM10* mutants, which correlated with an abnormally large morphology [37, 51]. Therefore, the larger colony size observed may be correlated to an increase in cell size within the population.

### Identification of QTL regions associated with susceptibility to antifungal agents

To identify the genomic regions underlying antifungal resistance in the hybrid progeny, we used the multipool approach [52] and sequenced a pool of the 20 best and of the 20 worst performing strains for three antifungal drugs acting on different biological processes, namely fluconazole, micafungin and flucytosine. The resulting genomic data was analysed following the hybrid QTL pipeline (Figure S1, https://github.com/Sookie-S/QTL_analysis_pipeline_hybrid_species/tree/main) which allowed the identification of QTL regions where allele frequencies significantly differed between the two pools of spores and exhibited a diametrically opposite trend. We have performed an independent analysis for each species sub-genome, obtaining all the *S. cerevisiae* QTLs (where the frequencies of the parental alleles from the *S. cerevisiae* strains differed between the pools) and all the *S. kudriavzevii* QTLs (where the frequencies of the parental alleles from the *S. kudriavzevii* strains differed between the pools).

Across the three drugs, a total of 101 and 89 different QTL regions were identified in the *S. cerevisiae* and in the *S. kudriavzevii* genomes, respectively (Table 2). These results reinforce the notion that while usually antifungals target a specific enzyme or metabolic process, the genetic background has a strong influence on the degree of drug resistance/susceptibility shown by the host. The largest number of QTLs was detected in flucytosine with 47 and 51 QTL regions identified in *S. cerevisiae* and *S. kudriavzevii*, respectively. The higher number of QTL associated with the trait may result from a higher complexity of the target of flucytosine, which is known to affect both RNA and DNA biosynthesis in fungi [10].

**Table 2:** Number and average length of QTL regions and genes identified in S*. cerevisiae* (Sc) and *S. kudriavzevii* (Sk) grown on different drugs

	**Number of QTL regions detected**		**Number of genes within the QTL regions**		**Average mean length of QTL regions**	
Genome	**Sc**	**Sk**	**Sc**	**Sk**	**Sc**	**Sk**
Micafungin	25	17	93	48	13.36 ± 11.08	16.31 ± 10.95
Fluconazole	29	21	212	107	18.85 ± 23.21	14.38 ± 18.17
Flucytosine	47	51	175	279	11.15 ± 13.84	15.46 ± 13.05
Total	101	89	480	434	-	-

Around 56% of genes identified within the QTL regions had one or more SNPs between the parental strains (Table S2). The SIFT (Sorting Intolerant from Tolerant) algorithm was used to identify non-synonymous SNPs between the two parental alleles of *S. cerevisiae* that could affect the protein function [48, 53].

### Identification of potential causal genes within QTL regions

A total of 136 genes (Table 3) have been identified in QTL regions that have potential to affect the phenotype based on previously published literature. Of the 136 genes, 75 were identified across QTL regions within either *Sc* and *Sk* genomes that have previously found to have a direct role in resistance to antifungal drugs (fluconazole, flucytosine, phleomycin, caspofungin and echinocandin) based on classical genetic [54] or transcriptome studies [16] in *S. cerevisiae* (Table 3). Fifty-six were found interacting with the drug target (*e.g.* with *ERG11* in fluconazole-QTLs) or with cellular processes closely linked to the drug mechanism of action (*e.g.* nucleotide biosynthetic processes for flucytosine-QTLs or β-glucan metabolism for micafungin-QTLs). Moreover, in micafungin-QTLs, we identified four orthologues of *Schizosaccharomyces pombe* genes associated with micafungin resistance [19]. Amongst these, *TOR1*, a protein kinase involved in signal transduction, cell growth and autophagy, was found similarly involved in the development of resistance to caffeine, a cell wall stressor, in both *S. cerevisiae* and *C. albicans* [20].

**Table 3:** List of 136 causal genes identified in QTL regions. Genes are clustered depending on their role or by the phenotype recorded in genetic studies. 75 genes are listed for their known role in resistance to antifungal drugs, further genes are listed for their role in pathways associated with antifungal drug susceptibility, including 30 genes with physical or genetic interactions with *ERG11*

**Causal genes identified in Fluconazole QTLs**		
	***S. cerevisiae* alleles**	***S. kudriavzevii* alleles**
**Increased resistance to fluconazole**	*PPZ1, SLY41, YAP1, ARP8, DEG1, HST4, ERV25, RAD17, ISW2, HAP5, ERG6, MRT4, SNU66, ADR1, GYP1, DST1, PSP2, MSA1, ANY1, NUP42, MRX16, TAF12, UBP6, CDC5, ITT1, UPC2, ALG8, PDE2, NDD1, COM2, HSC82, SWI5, COQ4, LDB19, VTS1, PRT1*	*SSK1, PUB1, RDH54, KES1, VPS27, CSE2, ACS2, ARK1, CBF2, HDA1, SOL1*
**Ergosterol biometabolism**	*ERG6, HEM2, UPC2*	*ARE2*
**Reported physical interaction with *ERG11***	*PPZ1, ERV25, ERG6, LAC1*	*ASI3, KES1*
**Reported genetic interaction with *ERG11***	*DOP1, TAF12, CBS2, SPC19, RAV2, RAD9, UBC6, RNA15, UBX2, SGS1, RPL36A, GYP1, HEM4, PLP2, DGK1, FAA1, PDE2, MRS6*	*NOC3, RPS10B, PET8, ASI3, LRO1, DBP6, PRP46*
**Causal genes identified in Flucytosine QTLs**		
	***S. cerevisiae* alleles**	***S. kudriavzevii* alleles**
**Resistance to flucytosine**	*BCH2, LSM6, ATO3, NKP1, ATP17, XRS2*	*PEX8, RRT2, BUB3, ROM1, NOC2, STP3, BFR1, MAK21, RRP46, PAC10, HMS1*
**DNA Damage Repair**	*RDH54, ESC2, POL1, RAD3, MLH1*	*DDR48, EXO1, IES4, MLH1*
**Resistance to phleomycin**	*CPR5, DNF1, HXK1, HNT3, BCK2, ENV11*	*SCL1, AIM34*
**Drug resistance**	*PDR15, RDS2*	*PDR10*
**Nucleotide biosynethetic processes**	*ADE8, ADK2*	*DUT1, ADE16, CDD1*
**DE genes and paralogs after flucytosine treatment in S. cerevisiae**	*RPL12B, ADE8, PDR15*	*MMT1, SPT21, CTL1, SER1, RPL9B, RPL15A*
**Causal genes identified in Micafungin QTLs**		
	***S. cerevisiae* alleles**	***S. kudriavzevii* alleles**
**Resistance to caspofungin**	*AKR1, MEH1*	*FEN2, MSN5, YSP2*
**Resistance to echinocandin**	*BCK2*	
**Beta glucan biometabolism**	*EXG2*	
**Cell wall stress**	*BCK2*	*RQC1, CNB1*
**Resistance to caffeine**	*IPK1, BCK2, TOR1*	*DPH2, DCG1, CNB1*
**Orthologues of *S. pombe* genes associated with micafungin resistance**	*MKC7, TOR1, HEL1, YTA6*	

Amongst the 83 potential causal genes identified from *S. cerevisiae*, SIFT analysis identified 11 alleles that carry SNPs predicted to cause a strong deleterious effect on the protein function (13.25%) while 38 were inferred to have tolerated mutations (45.8%) (Table S3).

A number of QTL regions encompassed genes that have not previously been associated with drug resistance or susceptibility. These can be genes that have an impact in the original strains/species and were not previously identified, or they could be more specifically associated with the hybrid background. In total we found 41 QTL regions containing genes not previously reported in the literature for both *S. cerevisiae* and *S. kudriavzevii* (Table S4, Table S5). A total of 23 genes were identified in high LOD intervals (*i.e.* > 16.0; Table 4), of which 11 were flagged in SIFT analysis as harbouring tolerated non-synonymous SNPs between the parental alleles, and among those, 3 were identified as harbouring potentially deleterious effects on protein function. One of such genes, identified within the *S. cerevisiae* QTL interval associated with fluconazole resistance (Table 4), is *NBP2,* a regulator of the high-osmolarity glycerol (HOG) pathway in yeast [55]. Nbp2p interacts with Bck1p and down-regulates the PKC-MAPK cell wall integrity pathway [56]. The PKC1-MAPK pathway has been previously identified as impacting fluconazole resistance in both *S. cerevisiae* and *C. albicans*. Specifically, the inhibition of PKC signalling prevents the activation of calcineurin, a key regulator of the membrane stress response, and hence raising the susceptibility to the antifungal [57, 58]. So, our data suggest that within the PKC1-MAPK pathway, the different *NBP2* alleles inherited in the hybrid background are important contributors to the phenotype in presence of fluconazole.

**Table 4:**
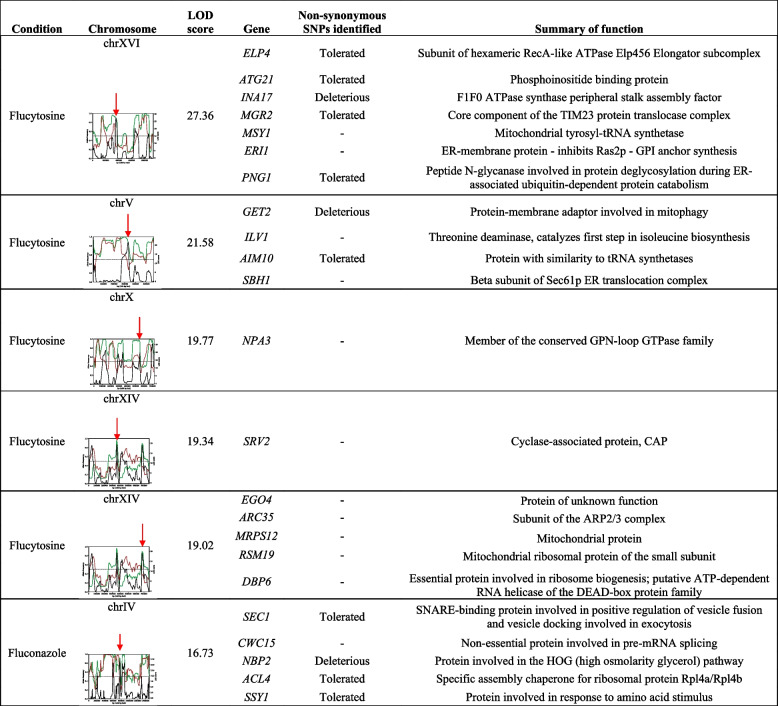
A summary of high LOD scoring intervals and the genes identified within the QTLs

QTL plots highlight the high LOD regions across the chromosome with a red arrow. The red and green line represent frequency of the alternative allele (ScOS253) in the high and low fitness pools respectively. The black line represents the LOD score across the plot

### Species overlap of QTL regions found only in flucytosine-QTLs

**Fig. 3:**
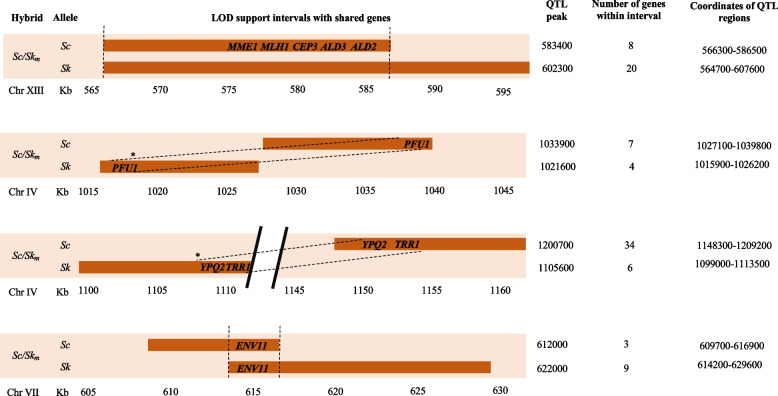
QTL intervals identified as significant for flucytosine susceptibility that are shared between *S. cerevisiae* and *S. kudriavzevii* genomes. For each interval shared, the position and the peak of the individual QTL is specified. Genes identified that are shared between *S. cerevisiae* and *S. kudriavzevii* are listed. *Regions containing shared genes in chromosome IV occupy different relative positions on the chromosomes of the two yeast species

Four QTL regions were identified in both *S. cerevisiae* and *S. kudriavzevii* alleles as affecting the hybrid fitness in flucytosine (Fig. 3), while no common regions were detected in fluconazole and micafungin-QTLs. Amongst the genes mapped in the shared flucytosine-QTLs we were able to identify potential causal genes (Table 2), as their function was linked to DNA synthesis and maintenance or to drug sensitivity in previously published work. In particular, *ENV11* and *TRR1* deletions were found to affect sensitivity to phleomycin [59, 60], while *ALD2* null mutants had increased sensitivity to floxuridine, a pyrimidine analogue which inhibits DNA and RNA synthesis similarly to flucytosine [61]. Moreover, *MLH1* is an ATPase involved in meiotic mismatch repair in mitosis and meiosis [62] while *PFU1* overexpression was found to exacerbate UV radiation toxicity in overexpression studies [63].

The limited overlap of QTL regions between species was also observed previously [33], and is somewhat surprising. It has been shown that in an inter-species background, the majority of genes preferentially express one parental allele over the other [64]. Thus, in an inter-species hybrid it is possible that the effect of allelic variation might be visible only for the species which is preferentially expressed and masked for the other. So, we can expect that a QTL study carried out separately in intra-species hybrid backgrounds, such as for *S. cerevisiae* hybrids (*Sc*^*OS104*^ x *Sc*^*OS253*^) and *S. kudriavzevii* hybrids (*Sk*^*IF01802*^ x *Sk *^*OS575*^), may uncover a greater overlap in QTL regions between the two species.

### Pleiotropic QTLs are rare across antifungal drugs

The difference in the mechanism of action of the fluconazole, micafungin and flucytosine resulted in a high number of trait-specific QTLs (99%), with only three pleiotropic regions, involving *S. cerevisiae* alleles, shared across conditions (Table 5). A small region in the chromosome V of *S. cerevisiae*, encoding for Pab1p, Bck2p and Dnf1p, was mapped in all three conditions. *BCK2* encodes a protein involved in the regulation of the cell cycle, and was previously shown to be important for growth in the presence of cell wall stressors (*i.e.* caffeine and echinocandin) and antifungal drugs, such as phleomycin, affecting DNA and RNA synthesis [59]. Here, we show that *BCK2* alleles are important for growth on three further drugs, supporting the pleiotropic nature of this gene.

**Table 5:** List of pleiotropic QTLs in *S. cerevisiae* x *S. kudriavzevii* hybrid diploid progeny. The coordinates of the pleiotropic intervals are specified along with the genome and the selection condition in which they were identified

*S. cerevisiae* genome	Chromosome	Start (bp)	End (bp)	Selection conditions	Genes in QTL interval
	IV	730300	740100	Fluconazole Micafungin	*MKC7, TAF12, SWI5, EKI1*
	V	506200	519700	Fluconazole Flucytosine Micafungin	*PAB1, DNF1, BCK2*
	XV	592700	602600	Fluconazole Flucytosine	*LSC1, THI80, ELG1, PNO1*

*DNF1,* is a flippase involved in the phospholipid translocation, and its deletion increase the fitness of *S. cerevisiae* grown on phleomycin [59]. Additionally, it has been shown that the deletion of *DNF1* orthologue in *Nakaseomyces glabratus* (formerly *Candida glabrata*) confers resistance to caspofungin [65]. Again, our data also highlight the pleiotropic nature of *DNF1*. Interestingly, *DNF1* and *BCK2* represent a super QTL in Sc/Sk hybrid background (a super-QTL is made up of linked genes that influence the same trait, these genes avoid segregation via recombination). Both alleles have a role in the antifungal resistance, and hence there may be an advantage in co-segregating them. Adaptation routes that aim to limit the segregation of advantageous allele combinations during recombination has been previously reported both in yeast (metabolic gene clusters) and in other organisms, such as ‘supergenes’ in butterflies [66].

### Validation of QTLs by Reciprocal hemizygosity analysis

**Fig. 4:**
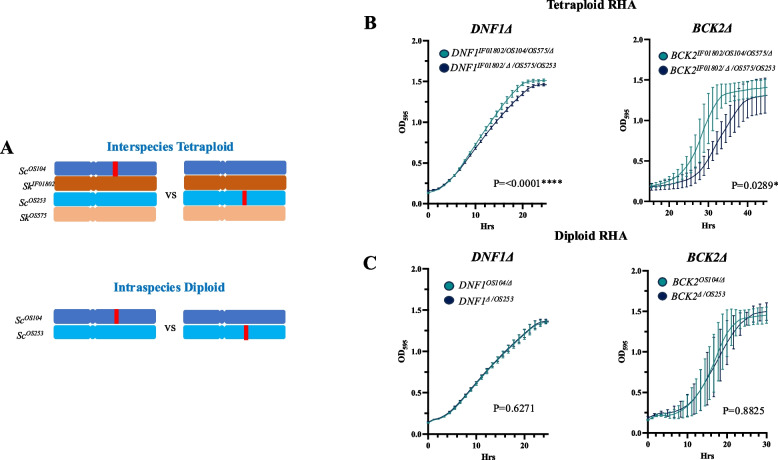
Growth curves for inter- and intra-specific hemizygotes assessed in antifungal drugs. **A** Diagram representing the process of reciprocal hemizygote generation for inter-species tetraploids, the GOI is knocked out via PCR-mediated deletion in the inter-species diploid. Mating with the WT diploid results in a hemizygote tetraploid for the Sc alleles. **B** Growth curves for Δ*DNF1* ScSkm tetraploid reciprocal hemizygotes and Δ*BCK2* ScSk_m_ tetraploid reciprocal hemizygotes **C**) Growth curves for the Δ*DNF1* ScSc diploids and the growth curve for the Δ*BCK2* ScSc diploids is shown. Growth assays were performed in YPD media + 50ug/mL flucytosine (*DNF1*) or YPD + 50ng/mL micafungin (*BCK2*) as highlighted in materials and methods. The integral area of the growth curves was calculated using the Growthcurver R package and significance assessed by the Student’s t-test (*P*>0.05)

We chose to validate two genes identified for their pleiotropic role, namely *BCK2* and *DNF1*, via reciprocal hemizygosity analysis (RHA) [49]. Dnf1p is a flippase involved in phospholipid translocation, and Bck2p is a protein involved in the G1/S transition of the cell cycle [67]. Both *DNF1* and *BCK2* harbour non-synonymous SNPs, with *DNF1* identified in SIFT analysis as containing potentially deleterious SNPs. The LOD score was highest for *DNF1* in flucytosine and *BCK2* in micafungin, therefore we chose to perform RHA for *DNF1* in flucytosine and perform RHA for *BCK2* in micafungin (Fig. 4A).

Given the complex genomic architecture of the hybrids, the RHA was carried out in liquid media as in previous studies [33] which allows for a greater discrimination between phenotypes compared to the solid media screening [68].

We created reciprocal allelic deletions of *DNF1* and *BCK2* (*i.e. Sc*^*D*^*/Sk*^*IF01802*^*/Sc*^*OS253*^*/Sk*^*OS575*^; *Sc*^*OS104*^*/Sk*^*IF01802*^*/Sc*^*D*^*/Sk*^*OS575*^) in the tetraploid parent (*Sc*^*OS104*^*/Sk*^*IF01802*^*/Sc*^*OS253*^*/Sk*^*OS575*^) and compared the growth of mutants in the presence of antifungal (Fig. 4B).

We observed a significant difference of the integral area under the growth curve for both *DNF1* and *BCK2* (Fig. 4B, Table S7). Moreover, there was a significant difference in maximum growth rate (*P* = 0.0001***) and final biomass (*P* = 0.0001***) between *DNF1* alleles in the presence of flucytosine (Fig. 4B, Table S7), and a significant difference in the lag phase (*P* = 0.0003***) for the *BCK2* alleles in response to micafungin (Fig. 4B, Table S7). The *DNF1*^OS104^ allele performed significantly better than the *DNF1*^OS253^ allele, mirroring the results of the QTL genetic screening. The two parental *BCK2* alleles also showed clearly different growth parameters, but it was the *BCK2*^OS104^ allele, prevalent in the low fitness pool, that performed better than *BCK2*^OS253^ with a significant difference in lag phase and integral area (Fig. 4B; Table S7). This discrepancy could be due the difference in phenotypic screening as the F12 progeny growth was scored on solid media, or to the effect of background as the F12 were diploid hybrids whilst the RHA was performed on the hemizygote tetraploids hybrids. Genetic interactions in tetraploids differ to the diploid progeny, differences in copy number and negative and positive epistasis can influence the phenotype [69]. Such transgressive segregation is widely observed in plants, where progeny show both fitter characteristics and less fit phenotypes than the parent [70]. The F12 tetraploids may demonstrate an altered phenotype in comparison to the diploid progeny as all alleles, both advantageous and deleterious, are present within one strain.

### Antifungal QTLs are specific to inter-species hybrids

To establish whether the QTLs identified are specific to the inter-species hybrid background, we tested the *BCK2* and *DNF1* allelic variants in two strains of the same species. We performed RHA using the parental *S. cerevisiae* strains *Sc*^*OS104*^ and *Sc*^*OS253*^, generating hemizygotic diploids (*Sc*^*OS104*^*/Sc*^D^ and *Sc*^D^/*Sc*^*OS253*^*)* with deletions of *BCK2* and *DNF1* alleles (Fig. 4A). Between allelic variants, we observed no significant difference in growth across both conditions (Fig. 4C, Table S8) with no significant difference in the integral area under the growth curve in both conditions (*P* > 0.05), or for final biomass (Fig. 4C, Table S8).

Through RHA, we were able to validate the phenotypic effect of the candidate genes and identify allelic variants that represent markers of antifungal resistance. As previously noted in Naseeb et al. 2021 [33], new and unique QTLs are identified in hybrids, we have confirmed that two genes identified in inter-species progeny do not have the same phenotypic effect in the *Sc/Sc* diploid background. Hybrid models, such as that developed by Timouma et al. for the hybrid *Saccharomyces pastorianus* [71], are needed to understand the emergence of allelic variants that impact phenotype exclusively in hybrids.

## Conclusions

This study allowed us to investigate how natural variation, developing outside clinical samples, may affect the evolutionary pathway leading to a resistant phenotype. Hybridisation in commonplace across yeasts and fungi including pathogenic genera, notably in *Candida* spp. and *Cryptococcus* spp. [27]. Inter-species hybridisation acts as a novel source for genetic variation and this process can lead to the development of strains with greater virulence than the parental species [24]. Here, we exploited recent advances in multigenerational breeding of *Saccharomyces* inter-species hybrids to study the impact of the allelic variants on the resistance and susceptibility to a diverse range of antifungal drugs, including azoles, pyrimidine analogues and echinocandins. We demonstrated the broad phenotypic diversity of the diploid hybrid progeny across the conditions tested and developed a robust pipeline for the identification of QTL regions in yeast inter-species hybrids. We identified major QTLs for the different drugs, including some not previously associated with antifungal resistance. Three, pleiotropic regions, involving *S. cerevisiae* alleles, were shared across all drug conditions. We validated, via reciprocal hemizygosity analysis, the impact of allele variants of two pleiotropic genes, namely *BCK2* and *DNF1*, in response to micafungin and flucytosine, respectively, and showed that the phenotype was hybrid specific. Interestingly, these two genes are linked in a “super QTL”, suggesting an evolutionary trajectory to limit the independent segregation of advantageous allele combinations [66]. Pleiotropy is not uncommon in markers of antifungal resistance; for example *PDR1* is well known for its role in multidrug resistance in *Saccharomyces* and *Candida* [72].

Understanding the unique genetic interactions underlying the phenotype observed in hybrid organisms will inform the design of personalised therapeutic pathways for the treatment of pathogenic hybrid yeasts. Furthermore, the dissection of pleiotropic QTLs could allow the identification of new predictors of drug resistance useful to tackle human pathogens.

## Supplementary Information

Supplementary Material 1

Supplementary Material 2

Supplementary Material 3

Supplementary Material 4

Supplementary Material 5

Supplementary Material 6

Supplementary Material 7

Supplementary Material 8

Supplementary Material 9

Supplementary Material 10

## Data Availability

Sequencing data have been deposited in the European Nucleotide Archive (https://www.ebi.ac.uk/ena/browser/view/PRJEB70903). All other study data are included in the article and/or supporting information.
