# Supplementary figures and images for: Impact of inter-species hybridisation on antifungal drug response in the Saccharomyces genus
Source: BMC Genomics. 2024 Dec 2;25:1165. doi: 10.1186/s12864-024-11009-3 (PMC11610120; doi:10.1186/s12864-024-11009-3)

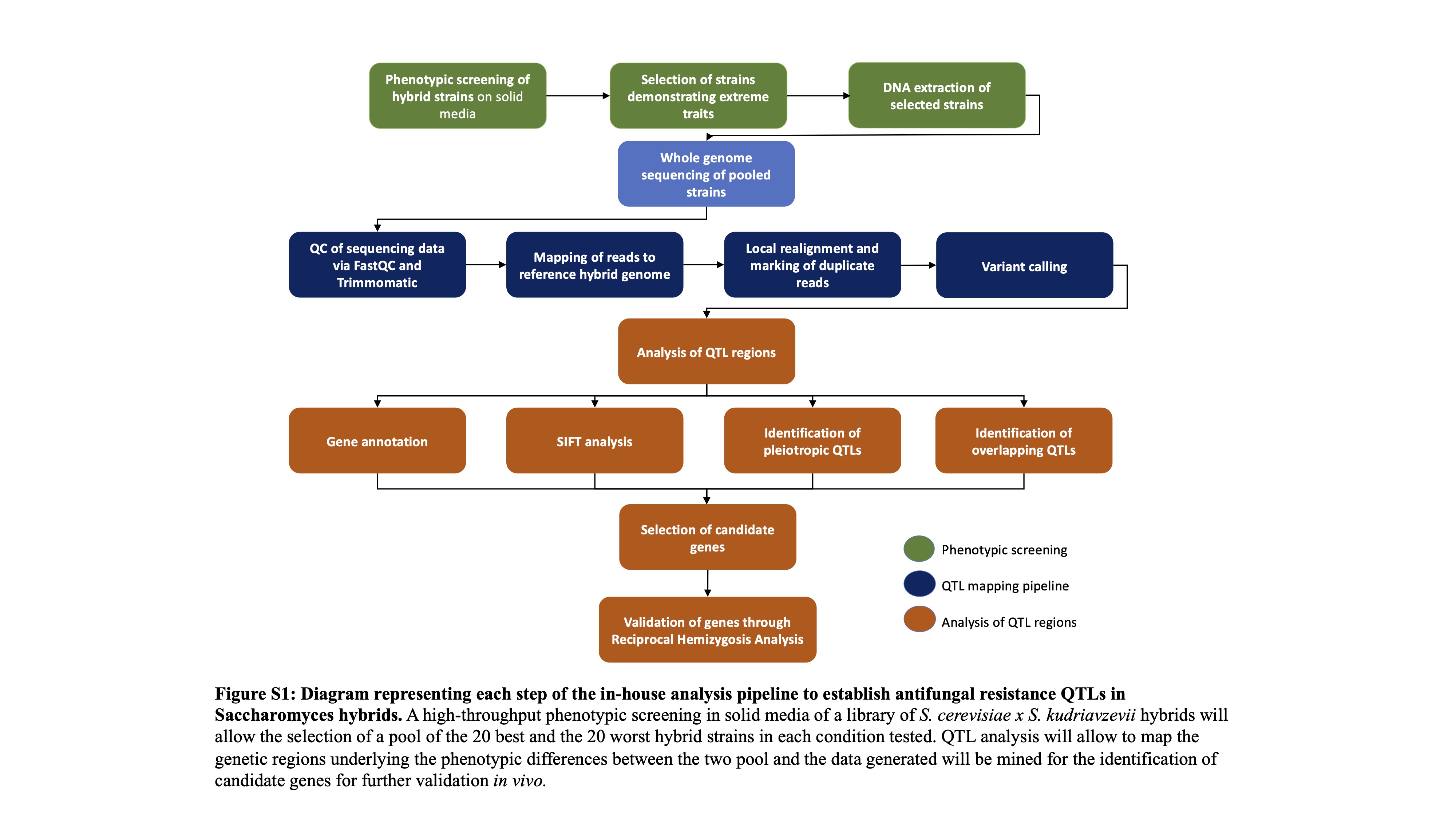

Supplement: Supplementary file 2 — Supplementary Material 2 [file 12864_2024_11009_MOESM2_ESM.jpg]

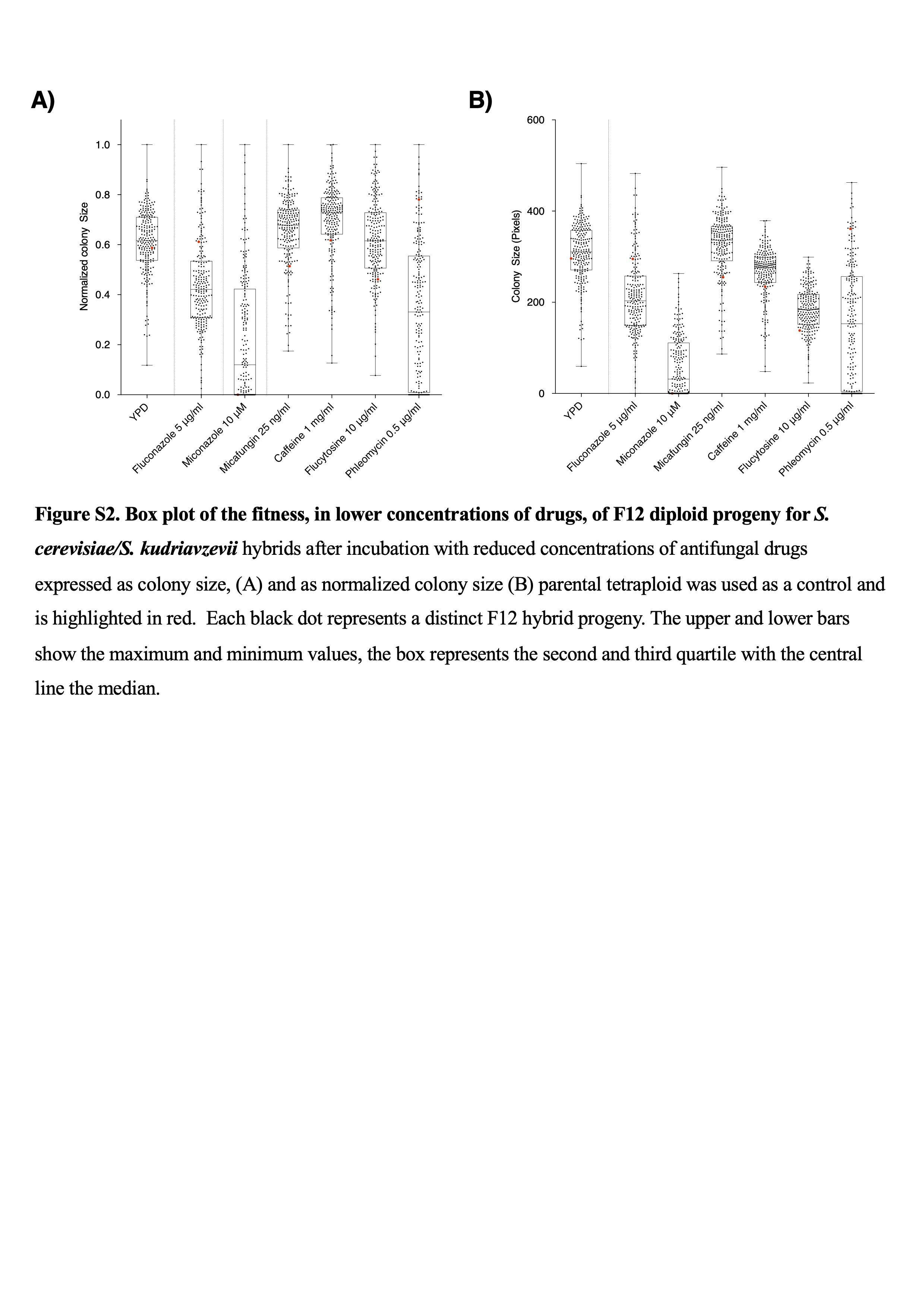

Supplement: Supplementary file 3 — Supplementary Material 3 [file 12864_2024_11009_MOESM3_ESM.jpg]
